# Late presentation of a paranasal sinus glass foreign body: a case report

**DOI:** 10.1186/1757-1626-2-6483

**Published:** 2009-05-29

**Authors:** Jitendra Mangwani, Archibald Paul Su

**Affiliations:** 1 Colchester General HospitalEssex, CO4 5JLUK; 2 ENT Department, Basildon HospitalEssex, SS16 5NLUK

## Abstract

Foreign bodies in the paranasal sinuses are rare and mostly related to maxillo-facial trauma. We treated a 47-year-old man with a late complication arising from a foreign body in the nasoethmoid sinus present for 16 years after a road traffic accident. Patients presenting with maxillo-facial injuries, especially those with lacerations due to glass or car wind-screen trauma should have thorough examination and appropriate imaging of the injury.

## Case presentation

A 47-year-old Caucasian man presented with forehead swelling and puffiness around the eyes. The swelling had gradually increased over a period of two months. The patient had purulent nasal discharge and no visual symptoms. Sixteen years ago, he was involved in a road traffic accident in which he sustained a laceration on his forehead as he collided with the windscreen of the car. He attended his local accident and emergency department and the laceration was treated by primary suturing under local anaesthesia (LA). No Plain radiographs were carried out. The wound healed well and the patient had no problems.

**Figure 1.**
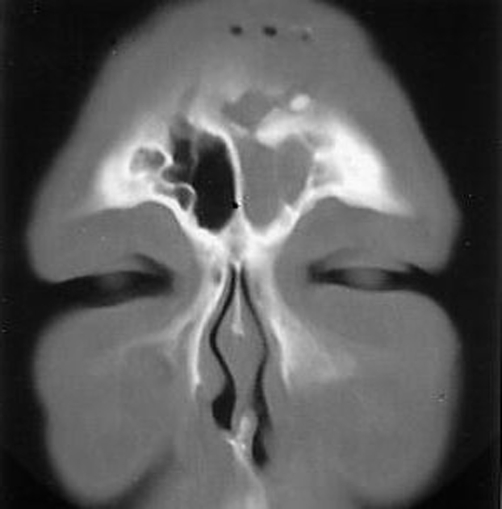
CT scan of the para nasal sinuses showing a left ethmoid sinus foreign body and opacification of all sinuses on the left.

On examination, a 5 cm linear scar was noted over the forehead swelling. The swelling measured 6 cm x 3 cm and was mildly tender. The patient underwent a complete ear nose throat examination. The nasal speculum examination showed sinusitis of the left paranasal sinuses which was confirmed by endoscopy. The CT (computed tomography) scan of paranasal sinuses (Figure 1) showed a radio-opaque foreign body (FB) in the left ethmoid sinus with opacification of all sinuses on the left side.

The patient underwent functional endoscopic sinus surgery with removal of glass fragment and draining of the sinuses. His symptoms resolved completely following the procedure. At 12 months follow-up, the patient has had no similar problems and remains asymptomatic.

## Discussion

Seventy percent of FBs in the paranasal sinuses are associated with some form of maxillofacial trauma [1,2]. If not suspected, they may escape detection during initial examination [3]. Fifty percent of such FBs are found in the maxillary sinus. They can also be found in other para-nasal sinuses and the reported incidence is equal [4]. Glass and metal fragments are the commonest forms of FBs seen within the para-nasal sinuses [5,6]. Although glass is inert, it can cause recurrent infection or obstruction of the frontonasal duct [7].

In the case reported, the patient presented with purulent left para-nasal sinusitis due to obstruction of the left frontonasal duct caused by an undetected glass fragment 16 years following the injury. To the best of our knowledge, such a late presentation of this complication has not been reported in the literature.

Patients with maxillo-facial injuries due to glass or car wind-screen trauma should have thorough examination of the wound. Simple lacerations can be inspected under LA but complicated injuries with deep maxillo-facial lacerations should have a wound inspection in the operating theatre.

Plain radiographs are useful to detect FBs [3]. Axial CT is the examination of choice for a suspected sinus FB. CT is helpful in localising the foreign body accurately, assesses the integrity of the anterior and posterior frontal sinus wall, the condition of mucosa, intracranial penetration and possible complications [2]. Endoscopic sinus surgery for removal of sinus FBs is effective and avoids the need for an external ethmoidectomy [4].

## Conclusion

This case illustrates that a high index of suspicion of a FB should be maintained in a patient with maxillo-facial trauma. A careful examination of the wound and appropriate imaging may help avoid future complications.

## Abbreviations

CT: computed tomography
LA: local anaesthesia
FB: foreign body
